# “STANDING IN THE GAP”: EXPERIENCES OF MEDICAID HOME AND COMMUNITY-BASED SERVICE SUPPORTS PLANNERS IN MARYLAND

**DOI:** 10.1093/geroni/igae098.1231

**Published:** 2024-12-31

**Authors:** Chanee Fabius, Sharmini Rathakrishnan

**Affiliations:** Johns Hopkins University, Baltimore, Maryland, United States; Johns Hopkins University, Baltimore, Maryland, United States

## Abstract

Medicaid Home and Community-Based Service (HCBS) care managers (called “supports planners” in Maryland) are responsible for coordinating care delivery and play a crucial role in bridging the gap between older adults, families, and home care and state agencies. This assistance is particularly relevant for older adults living with dementia and their families, as they represent nearly one-third of Medicaid HCBS participants, nationally. Despite their pivotal role in home care delivery and care quality, there is a paucity of research describing the contributions and experiences of supports planners. This study aims to improve the understanding of the role of supports planners, with implications for enhancing care coordination for older adults with dementia within Maryland’s Medicaid HCBS. We conducted seven semi-structured interviews with supports planners in Maryland from December 2023 to February 2024. Interviews were recorded, transcribed, and coded by the research team. Supports planners ranged in age from 26 to 54, were mostly female (n=5). Four supports planners were white, and three were black. Thematic analysis revealed four key themes highlighted by supports planners: 1) collaborations with paid and unpaid caregivers; 2) dementia-related care coordination; 3) the electronic management system as an information hub; and 4) supports planners’ role as liaisons in HCBS. The present study further emphasizes that supports planners are involved in numerous aspects of home care delivery. Our findings illuminate the role of supports planners in Medicaid HCBS and have implications for improving care coordination for older adults living with disabilities, especially those living with dementia.

